# Delayed neutrophil shedding of CD62L in patients with chronic rhinosinusitis with nasal polyps and asthma: Implications for *Staphylococcus aureus* colonization and corticosteroid treatment

**DOI:** 10.1002/clt2.12347

**Published:** 2024-03-10

**Authors:** Maryam Jafari, Eduardo I. Cardenas, Sandra Ekstedt, Julia Arebro, Marianne Petro, Agnetha Karlsson, Eric Hjalmarsson, Daniel Arnarson, Monika Ezerskyte, Susanna Kumlien Georén, Lars Olaf Cardell

**Affiliations:** ^1^ Division of ENT Diseases Department of Clinical Science, Intervention and Technology Karolinska Institutet Stockholm Sweden; ^2^ Department of ENT Diseases Karolinska University Hospital Stockholm Sweden

To the Editor:

Chronic rhinosinusitis with nasal polyps (CRSwNP) is an inflammatory disease of the sinonasal mucosa that is often accompanied by local *Staphylococcus aureus* colonization,^1^ as well as comorbid asthma.^2^ Although both CRSwNP and asthma are associated with a type 2 inflammatory profile, a growing body of literature indicates that neutrophils can also contribute to the pathophysiology of these diseases. For instance, shedding of CD62L (L‐selectin) is commonly used as a marker of neutrophil activation,^3^ and we have previously shown that neutrophils isolated from nasal polyps of patients with CRSwNP are characterized by low CD62L and high CD16 (FcγRIII) surface expression.^4^ Moreover, we have also shown that inhaled allergen provocation results in CD62L shedding in circulating neutrophils from patients with allergic asthma.^5^ However, the response of circulating neutrophils to bacterial stimuli has not been characterized in patients with both CRSwNP and asthma in comparison with healthy controls.

In this study, we isolated blood neutrophils from 19 patients with CRSwNP and comorbid asthma, as well as 20 healthy controls, and assessed their phenotype at baseline and in response to *S. aureus* enterotoxin A (SEA). A detailed description of the methods used can be found in Supporting Information S1, and the main characteristics of all study participants are summarized in Table 1. Freshly isolated blood neutrophils from healthy controls and patients with CRSwNP and comorbid asthma had a similar baseline surface expression of CD62L and CD16 (Figure S1), which confirms our previous findings in other cohorts of patients with CRSwNP or asthma.^4^, ^5^ Notably, most patients included in our study received inhaled corticosteroids (ICS) (Table S1), and a previous study suggests that ICS can impact the baseline surface expression of CD62L in unstimulated neutrophils.^6^ Nevertheless, the study by Pasternak et al. also determined that ICS have no impact on CD62L expression when administered via active inhaler, and our patients received ICS exclusively via active inhaler.

**TABLE 1:** Patient characteristics.

	Healthy controls (*n* = 20)	Patients with CRSwNP (*n* = 19)
Females/males (*n*)	16/4	8/11
Age (years)	45 (25–67)^a^	56 (20–77)^a^
Polyp removal surgeries (*n*)	N/A	1 (0–6)^a^
Comorbidities, *n* (%)	N/A	
Asthma		19 (100%)
Hypertension		9 (47%)
NSAID hypersensitivity		2 (10%)

Abbreviations: N/A, not applicable; NSAID, nonsteroidal anti‐inflammatory drugs.

^a^ Median (range).

Interestingly, in vitro stimulation with SEA for 2 h resulted in a marked decrease in CD62L surface expression in neutrophils from healthy controls, but not in neutrophils from patients with CRSwNP and asthma (Figure 1A,B). No significant changes in the neutrophil activation markers CD11b, CD66b or IL‐1β were detected in either study group at this timepoint (Figure S2). Nevertheless, neutrophils from both study groups had a similar decrease in CD62L surface expression 4 h post‐stimulation (Figure 1C,D), as well as a similar increase in CD66b surface expression at this timepoint (Figure S3A–C). In addition, neutrophils from healthy controls had an increase in CD11b and IL‐1β expression 4 h post‐stimulation, while neutrophils from patients with CRSwNP and asthma displayed a similar pattern that nonetheless failed to reach statistical significance (Figure S3D–I). Finally, the change in surface expression of CD62L between stimulated and unstimulated neutrophils was markedly smaller in neutrophils from patients with CRSwNP and asthma than in healthy controls at the 2‐h timepoint (Figure 1E,F), but similar between groups at the 4‐h timepoint (Figure 1G).

**FIGURE 1:**
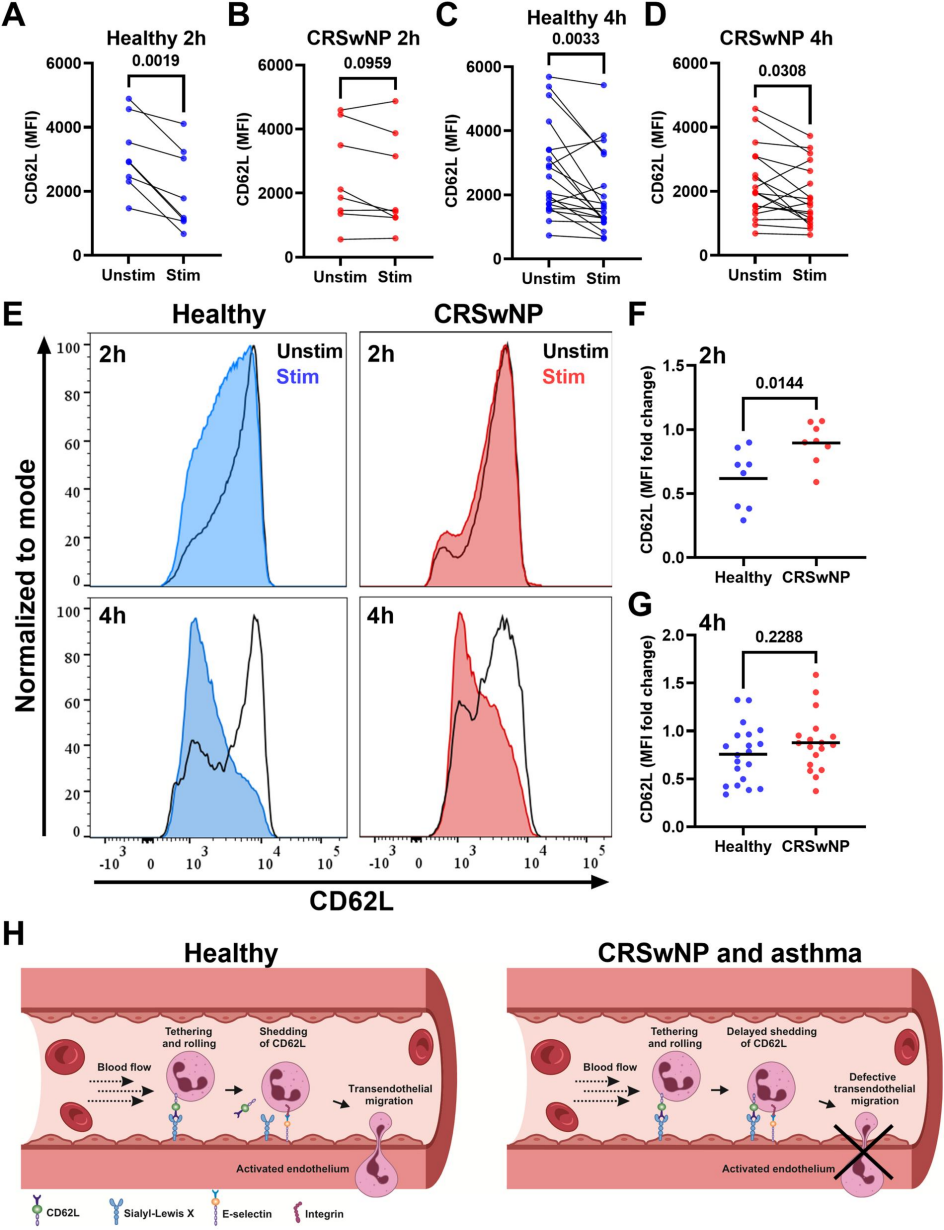
Neutrophil shedding of CD62L is delayed in patients with CRSwNP and asthma. Blood neutrophils from healthy controls (blue) and patients with CRSwNP and asthma (red) were stimulated with Staphylococcal enterotoxin A for 2 or 4 h. (A–D) Comparisons of the MFI of CD62L between unstimulated and stimulated neutrophils by paired Student's *t*‐test. (E) Representative histograms of CD62L MFI in unstimulated (black outline) and stimulated (blue/red) neutrophils. (F, G) Comparisons of the change in MFI of CD62L by unpaired Student's *t*‐test. *N* = 8 and 18 for the 2‐ and 4‐h timepoints, respectively. (H) Graphic summary of conclusions. CRSwNP, chronic rhinosinusitis with nasal polyps; MFI, mean fluorescent intensity.

In conclusion, our study revealed a delay in CD62L shedding in blood neutrophils from patients with CRSwNP and comorbid asthma, two conditions associated with type 2 inflammation, in response to a relevant bacterial stimulus. In addition, neutrophils from patients with CRSwNP and asthma had an attenuated expression of the activation markers CD11b and IL‐1β upon stimulation. CD62L mediates the initial attachment of neutrophils to activated endothelium, and its subsequent shedding is necessary for the optimal transendothelial migration (TEM) of neutrophils at sites of inflammation or infection (Figure 1H). Notably, prior research involving human neutrophils and genetically engineered mice has demonstrated that hindering CD62L shedding can impede neutrophil TEM in response to chemoattractants.^7^, ^8^ Therefore, our findings suggest that blood neutrophils from patients with CRSwNP and comorbid asthma might possess an impaired migratory phenotype which, combined with an attenuated activation potential, could contribute to the high *S. aureus* colonization rates that characterize CRSwNP. This highlights the relevance of neutrophils—more often linked to type 1 inflammatory conditions—in CRSwNP and asthma. Moreover, addition of corticosteroids to neutrophil cultures is known to impair the shedding of CD62L upon stimulation,^9^ and thus it seems likely that corticosteroid treatment in patients with CRSwNP and asthma may exacerbate the defect in CD62L shedding that we now report. Nevertheless, further research is needed to establish whether defective shedding of CD62L impacts the immune response to bacterial infections in patients with CRSwNP and comorbid asthma.

## AUTHOR CONTRIBUTIONS

Maryam Jafari, Sandra Ekstedt, Eric Hjalmarsson, Monika Ezerskyte, Susanna Kumlien Georén, and Lars Olaf Cardell designed the outline of the study. Maryam Jafari, Julia Arebro, Marianne Petro, Agnetha Karlsson, and Daniel Arnarson collected the patient material. Maryam Jafari, Sandra Ekstedt, and Marianne Petro performed the neutrophil stimulation and flow cytometry evaluation. Maryam Jafari and Eduardo I. Cardenas analyzed and compiled the collected data and performed all statistical analyses. Maryam Jafari, Eduardo I. Cardenas, Eric Hjalmarsson, and Lars Olaf Cardell drafted the original manuscript that was read and reviewed by all authors. All authors contributed to the article and approved the submitted version.

## CONFLICT OF INTEREST STATEMENT

The authors declare no conflict of interest.

## FUNDING INFORMATION

Sanof‐Genzyme Type 2 Innovation Grant

## Supporting information

Supporting Information S1

## Data Availability

The datasets that support the findings of this study are available from the corresponding author upon reasonable request.
