# Supplementary material for: Delayed neutrophil shedding of CD62L in patients with chronic rhinosinusitis with nasal polyps and asthma: Implications for Staphylococcus aureus colonization and corticosteroid treatment
Source: Clin Transl Allergy. 2024 Mar 10;14(3):e12347. doi: 10.1002/clt2.12347 (PMC10925549; doi:10.1002/clt2.12347)
Supplement: Supplementary file 1 — Supporting Information S1 [file CLT2-14-e12347-s001.docx]

**MATERIALS AND METHODS**

***Study material***

Nineteen patients with chronic rhinosinusitis with nasal polyps (CRSwNP; defined by EPOS^1^ [European Position Paper on Rhinosinusitis and Nasal Polyps] criteria based on symptoms, results of nasal endoscopy and computed tomography of the sinuses) and comorbid asthma were recruited at the Department of Ear, Nose and Throat Diseases of the Karolinska University Hospital in Stockholm, Sweden. In addition, 20 healthy controls with no history of sinus disease, asthma or allergy were recruited. Patients and healthy controls reported no acute infections three weeks prior to inclusion. Blood samples were collected at inclusion.

***Ethics Statement***

All participants provided written informed consent before inclusion and all procedures were conducted in accordance with the declaration of Helsinki and the Swedish Ethical Review Authority in Stockholm, Sweden (Diary No. 2015/295-31/2 and 2017/686-32).

***Neutrophil isolation and stimulation***

Neutrophils were isolated from heparinized whole blood using the MACSxpress Whole Blood Neutrophil Isolation kit (Miltenyi Biotec, Bergisch Gladbach, Germany). In brief, neutrophils were isolated via negative selection using antibody-conjugated magnetic beads that recognize surface antigens on all blood cells except neutrophils. Following magnetic removal of all non-target cells, untouched neutrophils were pipetted into a separate tube and residual erythrocytes were depleted as described above using the MACSxpress Erythrocyte Depletion kit (Miltenyi Biotec). Neutrophils were centrifuged (400 RCF, 5 min) and resuspended RPMI-1640 containing 0.2% penicillin-streptomycin (both from Gibco, Waltham, MA) and 10% participant-specific plasma. Resuspended neutrophils were seeded in 96-well plates (1x10^6^ cells/well) and stimulated (2 or 4 h, 37°C, 5% CO_2_) with 10 µg/ml Staphylococcal enterotoxin A (Sigma-Aldrich) or left unstimulated. Of note, neutrophils from 8 healthy controls and 7 patients with CRSwNP and asthma were included in both timepoints, neutrophils from 1 patient with CRSwNP and asthma were only included in the 2-hour timepoint, and neutrophils from 12 healthy controls and 11 patients with CRSwNP and asthma were only included in the 4-hour timepoint.

***Flow cytometry***

Stimulated and unstimulated neutrophils were Fc-blocked (5 min, room temperature; BD Biosciences, Franklin Lakes, NJ) and stained (20 min, room temperature) with the antibodies listed in Supplementary Table 2 except anti-IL-1β. Surface-stained neutrophils were fixed and permeabilized using the IntraPrep Permeabilization kit (Beckman Coulter, Indianapolis, IN) and stained intracellularly with anti-IL-1β (20 min, room temperature; Supplementary Table 1). Flow cytometry was performed on an LSRFortessa™ and data was analysed using FlowJo™ (both from BD Biosciences).

***Statistical analysis***

Comparisons of two normally distributed groups were performed via paired or unpaired Student’s t-test as specified in figure legends. Comparisons of two non-normally distributed and unpaired groups were performed via non-parametric Mann-Whitney test. All statistical analyses were conducted in Prism 9.4 (GraphPad, San Diego, CA) and statistical significance was defined as p < 0.05.

**REFERENCE**

1. Fokkens WJ, Lund VJ, Mullol J, et al. European Position Paper on Rhinosinusitis and Nasal Polyps 2012. *Rhinol Suppl.* 2012(23):3 p preceding table of contents, 1-298.

***Supplementary Table 1. Medications of study participants.***

|  | **Healthy Controls** | **Patients with CRSwNP**  **(n: active ingredient)** |
| --- | --- | --- |
| Nasal corticosteroids | N/A | 9: fluticasone propionate  8: mometasone |
| Inhaled corticosteroids | N/A | 8: budesonide  5: beclometasone  3: fluticasone propionate  2: ciclesonide |
| Long-acting bronchodilators | N/A | 14: formoterol  3: tiotropium bromide  1: theophylline |
| Short-acting bronchodilators | N/A | 6: salbutamol  3: terbutanile  1: ipratropium bromide |
| Antihistamines | N/A | 9: desloratadine  5: azelastine  1: propiomazine^1^ |
| Leukotriene receptor antagonists | N/A | 8: montelukast |
| Other | N/A | 5: amlodipine^2^  5: enalapril^3^  2: atorvastatin^4^  2: sertraline^5^  1: acetylcysteine^6^ |

Notes: N/A = Not applicable. ^1^sleep medication, ^2-3^hypertension medications, ^4^cholesterol-lowering medication, ^5^anti-depressant, and ^6^mucolytic.

***Supplementary Table 2. Antibodies used.***

| **Target molecule** | **Fluorochrome** | **Clone** | **Company** |
| --- | --- | --- | --- |
| CD11b | BUV805 | D12 | BD Biosciences |
| CD16 | V450 | 3G8 | BD Biosciences |
| CD62L | BV510 | DREG-56 | BD Biosciences |
| CD66b | PC-CY7/APC-Vio77 | REA306 | Miltenyi Biotec |
| IL-1β | APC | REA1172 | Miltenyi Biotec |


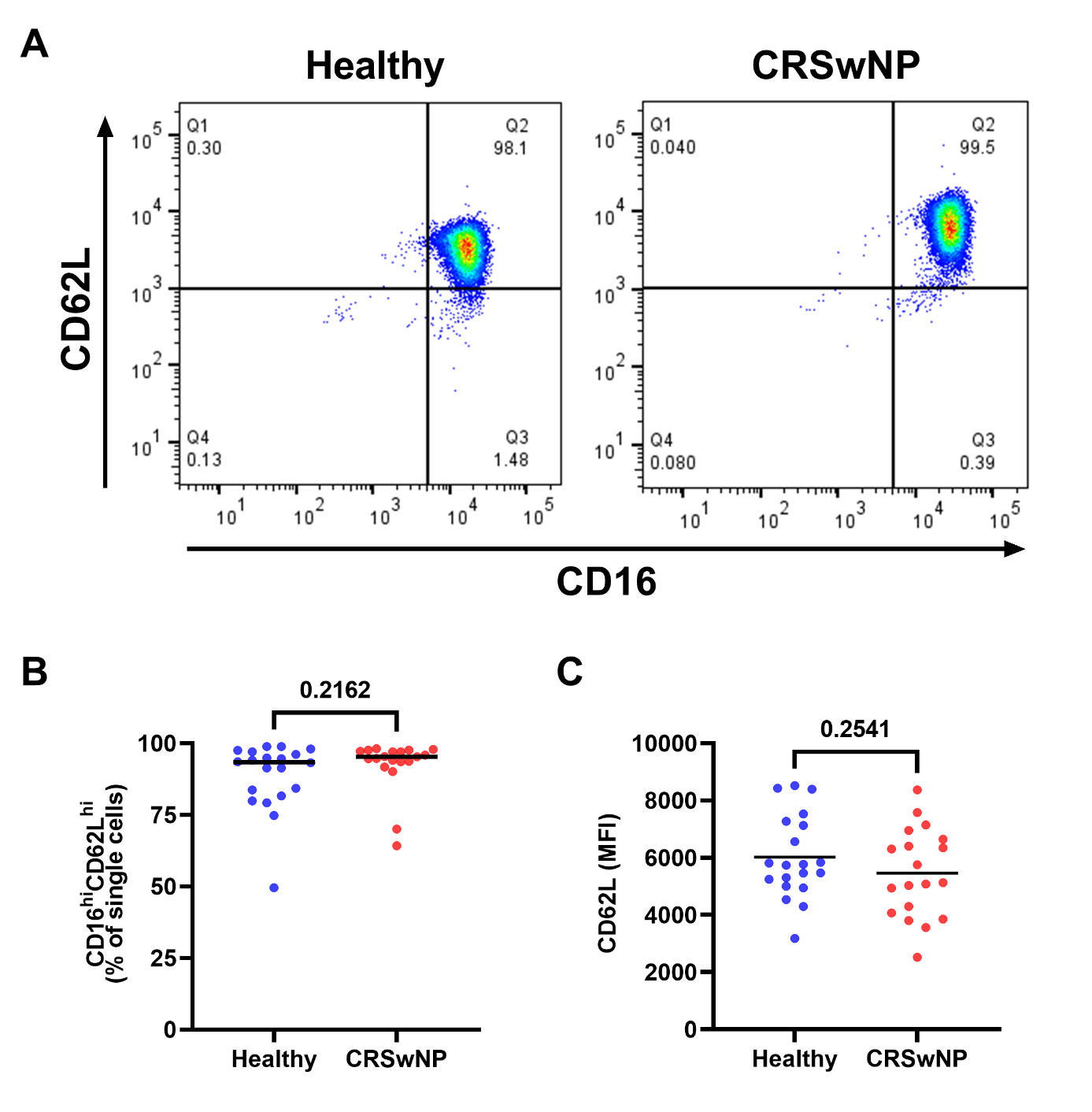


**Supplementary Figure 1. Circulating neutrophils from healthy controls and patients with CRSwNP and comorbid asthma have comparable expression of CD62L and CD16 at rest.** Blood neutrophils were isolated from healthy controls (n = 20) and patients with CRSwNP and comorbid asthma (n = 19) and stained for CD62L and CD16. **(A)** Representative expression of CD62L and CD16 in neutrophils at rest. **(B)** Comparison of the frequency of CD16^hi^CD62L^hi^ neutrophils at rest by Mann-Whitney test. **(C)** Comparison of mean fluorescence intensity (MFI) of CD62L in neutrophils at rest by unpaired Student’s t-test. Horizontal lines represent **(B)** median or **(C)** mean.


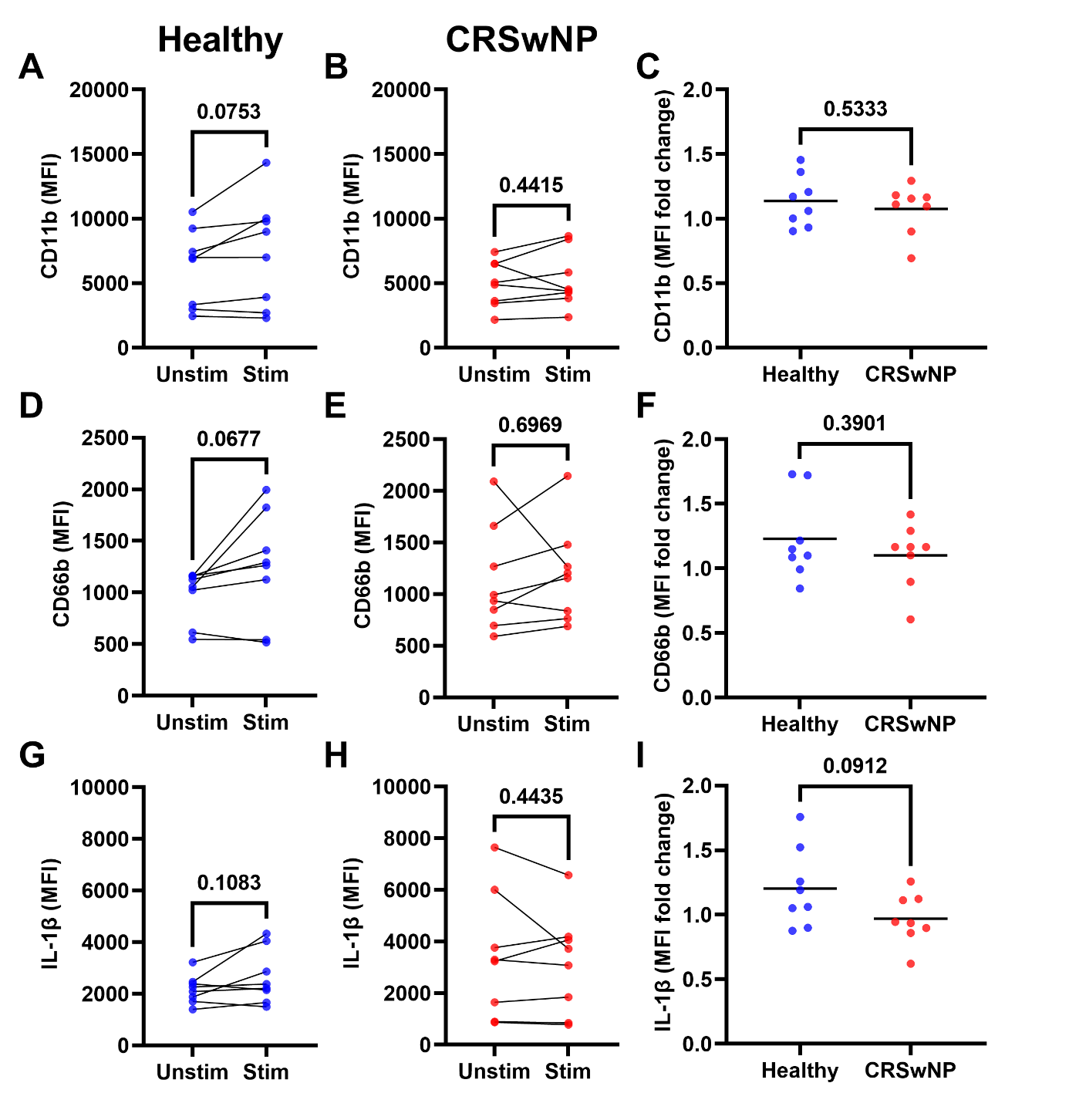


**Supplementary Figure 2. No statistically significant changes in CD11b, CD66b or IL-1β expression in neutrophils stimulated with Staphylococcal enterotoxin A for 2 hours.** Blood neutrophils were isolated from healthy controls (blue, n = 8) and patients with CRSwNP and comorbid asthma (red, n = 8) and stimulated with Staphylococcal enterotoxin A for 2 hours. Comparisons of the mean fluorescence intensity (MFI) of **(A-B)** CD11b, **(D-E)** CD66b, and **(G-H)** IL-1β in unstimulated (unstim) and stimulated (stim) neutrophils by paired Student’s t-test. Dots joined by a line represent single study participants. Comparisons of the change in MFI of **(C)** CD11b, **(F)** CD66b, and **(I)** IL-1β upon stimulation by unpaired Student’s t-test. Horizontal lines represent mean.


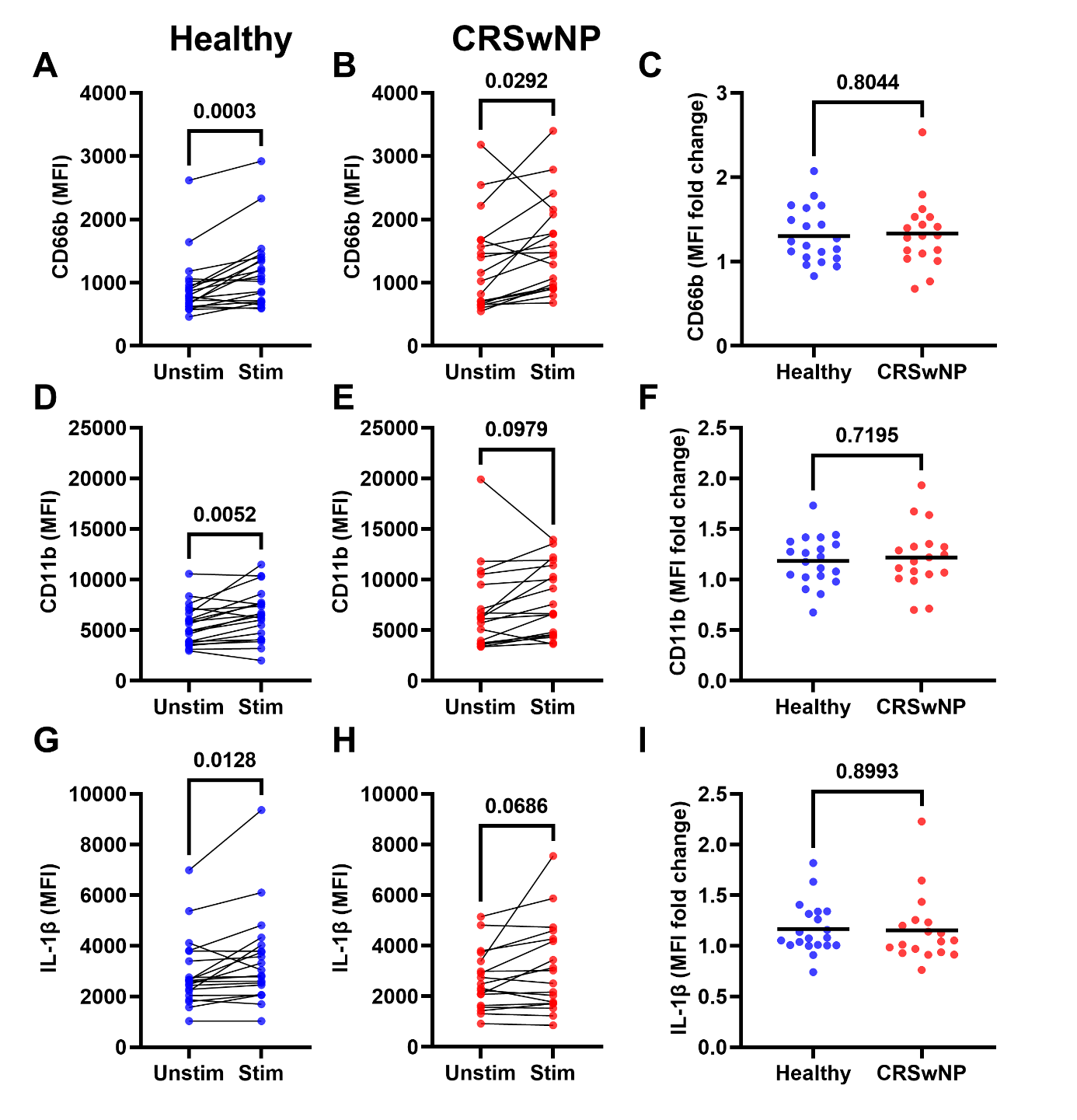


**Supplementary Figure 3. Neutrophils from healthy controls and patients with CRSwNP and asthma have a similar change in CD11b, CD66b and IL-1β expression upon stimulation with Staphylococcal enterotoxin A for 4 hours.** Blood neutrophils were isolated from healthy controls (blue, n = 20) and patients with CRSwNP and comorbid asthma (red, n = 18) and stimulated with Staphylococcal enterotoxin A for 4 hours. Comparisons of the mean fluorescence intensity (MFI) of **(A-B)** CD66b, **(D-E)** CD11b, and **(G-H)** IL-1β in unstimulated (unstim) and stimulated (stim) neutrophils by paired Student’s t-test. Dots joined by a line represent single study participants. Comparisons of the change in MFI of **(C)** CD66b, **(F)** CD11b, and **(I)** IL-1β by unpaired Student’s t-test. Horizontal lines represent mean.
